# Regional differences in personalities account for substantial heterogeneity of loneliness change from before to during the COVID-19

**DOI:** 10.3389/fpsyg.2023.1124627

**Published:** 2023-05-05

**Authors:** Shanshan Xiao, Junhua Dang

**Affiliations:** ^1^Department of Psychology, Stockholm University, Stockholm, Sweden; ^2^School of Education, Huaibei Normal University, Huaibei, Anhui, China; ^3^Department of Surgical Sciences, Uppsala University, Uppsala, Sweden

**Keywords:** personality, loneliness, COVID-19, meta-analysis, heterogeneity

## Introduction

Ernst et al. (2022) recently reported a strictly conducted meta-analysis investigating whether people's loneliness increased during the COVID-19 pandemic relative to prepandemic times. Based on 19 longitudinal studies that tracked the change of participants' loneliness scores, their main analysis found a small effect size, Hedge's *g* = 0.23 (see Supplementary Table S1 for a summary of the main information extracted from these articles). However, the heterogeneity between individual studies was very high, *I*^2^ = 98%, which means most differences observed between individual studies were due to real differences in effect sizes rather than random sampling errors. Although a series of factors have been considered (e.g., sample type and age) to account for the between-effects heterogeneity, none of them showed significant moderation, leaving the heterogeneity unanswered. Given the included studies were conducted in different countries, in this article we suggest that regional differences in the Big-Five personalities should be considered because loneliness has been strongly associated with personalities (Buecker et al., 2020) and people's personalities vary across countries (McCrae, 2001). Therefore, we test whether regional differences in the Big-Five personalities could account for the observed between-effects heterogeneity in Ernst et al.'s (2022) meta-analysis.

## Analysis

First, we used regional aggregated personality scores to indicate regional differences in personalities. The IPIP-NEO-300 is a 300-item representation of the NEO Personality Inventory (NEO-PI-R, Costa and MaCrae, 1992), which is called a “gold-standard” measure of the Five-Factor Model of personality (Johnson, 2017). The IPIP-NEO-300 is freely accessible and has been translated into multiple languages and extensively tested around the world via the Internet (Goldberg et al., 2006). John A. Johnson provided access to the scores of the five personalities collected from 307,313 people from different countries (https://github.com/automoto/big-five-data). Based on this, we calculated the mean score of each personality for regions/countries included in Ernst et al.'s (2022) meta-analysis, which is used to represent regional difference in each personality.

**Figure 1:**
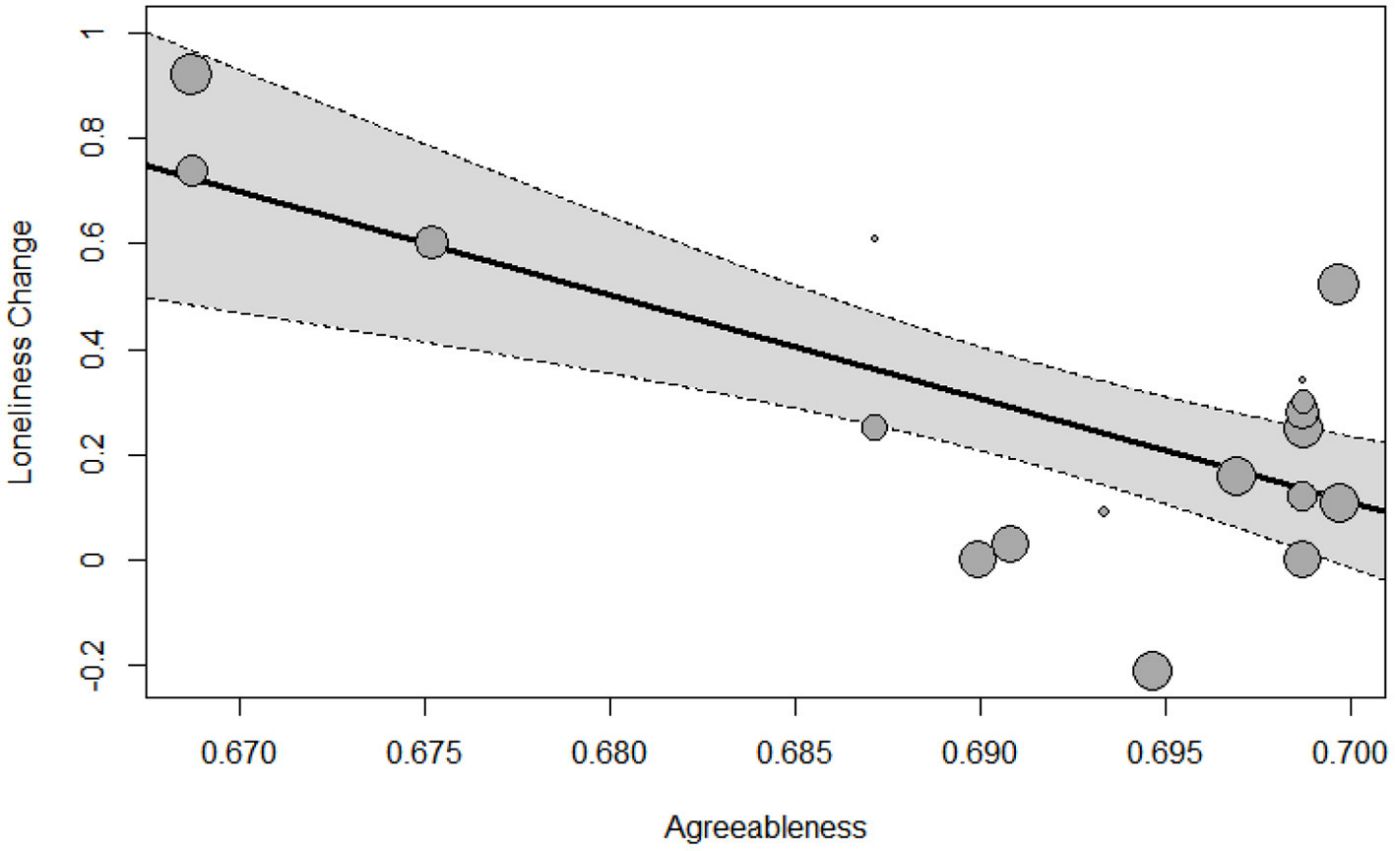
The effect size of loneliness change plotted against regional difference in agreeableness with corresponding 95% confidence interval bounds. The size of the points is drawn proportional to the weight that the studies received in the analysis (with larger points for studies that received more weight).

Next, we conducted univariate moderator analysis by testing regional differences in the five personalities one-by-one by using the metafor package in *R* software. The data and the *R* script can be found via this link (https://osf.io/f7ucb/?view_only=d99ed5ce0ed641ca80965e54c11afc81). As shown in Supplementary Table S2, regional difference in agreeableness was significantly associated with the effect size of loneliness change, *B* = −19.63, *p* < 0.001, which noticeably accounted for 46.97% of the between-effects heterogeneity. Figure 1 illustrates the scatter plot of this relationship, showing that longitudinal studies reported increased loneliness from before the pandemic to during the pandemic in regions where most people score lower in agreeableness. In contrast, in regions where most people score higher in agreeableness, longitudinal studies found negligible change of loneliness.

Finally, we conducted multivariate moderator analyses by including all five personalities in a single model to test for robustness. As shown in Supplementary Table S3, similar to univariate moderator analysis, agreeableness was negatively associated with the effect size of loneliness change, *B* = −36.17, *p* < 0.001. Moreover, extraversion was positively associated with loneliness change, *B* = 15.64, *p* = 0.005, which means longitudinal studies found increased loneliness in regions where most people score higher in extraversion. In total, the five personalities accounted for 65.22% of the between-effects heterogeneity, *Q*_*m*_(5) = 35.55, *p* < 0.001. Note that there were two studies that used the 20-item UCLA loneliness scale. Recently, it has been shown that the 20-item UCLA loneliness scale had a poor and/or inconsistent structure compared with its shorter versions (e.g., Panayiotou et al., 2022). Therefore, we repeated the above analyses by excluding the two studies that used the 20-item UCLA loneliness scale. The results were almost the same.

## Discussion

The moderating effect of regional difference in agreeableness was very robust, which survived in both univariate and multivariate analyses, showing that people's loneliness did not increase if they live in a region where most people are agreeable. In univariate moderate analysis, it alone accounted for 46.97% of the between-effects heterogeneity. This finding may be related to how people perceive the measures implemented by governments to arrest the rapid spreading of the virus. Agreeableness describes a person's tendency to put others' needs before their own. Those who are more agreeable are more likely to be empathetic and find pleasure in helping others (Costa and MaCrae, 1992). It has been found that empathy for people most vulnerable to the virus promoted acceptance of lockdown measures (Petrocchi et al., 2021) and the motivation to adhere to preventive guidelines (e.g., physical distancing) (Pfattheicher et al., 2020). It is likely that in regions reporting higher aggregated agreeableness, people were more likely to accept the stringent policies because of their empathy for others (especially the most vulnerable), which relieved perceived loneliness.

The moderating effect of regional difference in extraversion was significant in multivariate analysis, showing that people's loneliness increased if they live in a region where most people are extravert. Extraversion refers to how energetic, sociable, and friendly a person is Costa and MaCrae (1992). Preventive measures restricted people's activities and mobilities to a great extent, which is a particular load for extravert people. Therefore, it is likely that in regions with higher aggregated extraversion, people tend to feel higher loneliness during the pandemic relative to prepandemic times. However, this finding needs to be interpreted with caution because the moderating effect of extraversion was not significant in univariate analysis.

In conclusion, we suggest that regional differences in personalities may moderate the change of loneliness from before the pandemic to during the pandemic to a great extent. Our findings are not only useful for studies conducted during the COVID-19 pandemic but also helpful for research on global crises in the future.

## Author contributions

SX and JD contributed to the conception and design of the study. JD performed the statistical analysis and discussed with SX. SX wrote the manuscript and discussed with JD. Both authors contributed to the manuscript revision and approved the submitted version.
