# Supplementary material for: Regional differences in personalities account for substantial heterogeneity of loneliness change from before to during the COVID-19
Source: Front Psychol. 2023 May 5;14:1124627. doi: 10.3389/fpsyg.2023.1124627 (PMC10198375; doi:10.3389/fpsyg.2023.1124627)
Supplement: Supplementary file 1 [file Table_1.DOCX]

**Supplemental Materials**

Table 1. Studies included in Ernst et al.’s (2022) meta-analysis.

| Authors | Loneliness measure | Country | Sample (*N*) | Mean age | Women (%) | Effect size (*g*) |
| --- | --- | --- | --- | --- | --- | --- |
| Bartrés-Faz et al. (2021) | UCLA 3-item | Spain | 1604 | 55.7 | 0.65 | -0.21 |
| Bierman and Schieman (2020) | Single item | Canada | 2446 | 41.97 | 0.487 | 0.16 |
| Elmer et al. (2020) | UCLA 9-item | Switzerland | 212 | -- | -- | 0.25 |
| Entringer and Gosling (2021) | UCLA 3-item | Germany | 6010 | 52.64 | 0.6093 | 0.92 |
| Herrera et al. (2021) | UCLA 3-item | Chile | 721 | 72 | 0.6976 | 0.03 |
| Kivi et al. (2021) | UCLA 4-item | Sweden | 1071 | 67.13 | 47.49 | 0 |
| Kovacs et al. (2021) | UCLA 20-item | USA | 189 | 27.6 | 0.38 | 0.3 |
| Krendl and Perry (2021) | UCLA 3-item | USA | 93 | 75.2 | 0.543 | 0.34 |
| Lau et al. (2021) | UCLA 20-item | UK | 99 | 21 | 50 | 0.09 |
| Macdonald and Hulur (2021) | Single item | Switzerland | 99 | 71.49 | 0.374 | 0.61 |
| Mueller et al. (2021) | UCLA 3-item | USA | 280 | 12.4 | 0.65 | 0.12 |
| Pan et al. (2021) | JDJG 6-item | Netherlands | 1517 | -- | 0.64 | 0.11 |
| Peng and Roth (2021) | UCLA 11-item | USA | 1141 | 63.12 | 0.53 | 0 |
| Philpot et al. (2021) | NIH 5-item | USA | 1996 | 60 | 0.5 | 0.25 |
| Rogers et al. (2021) | UCLA 3-item | USA | 407 | 15.5 | 0.499 | 0.28 |
| van der Velden et al. (2021) | JDJG 6-item | Netherlands | 4084 | 45 | 0.507 | 0.52 |
| van Tilburg et al. (2020) | JDJG 6-item | Netherlands | 1502 | 72 | 0.49 | 0.11 |
| Werner et al. (2021) | UCLA 3-item | Germany | 443 | 22.8 | 0.77 | 0.74 |
| Wong et al. (2020) | JDJG 6-item | Hong Kong | 583 | 70 | 0.726 | 0.6 |

Table 2. Univariate moderator analysis by testing the five personalities one-by-one.

| Moderator | *Q_m_* | *B (SE)* | *p* | *R*^2^ |
| --- | --- | --- | --- | --- |
| Extraversion | 1.50 | -6.49 (5.30) | .221 | 2.50% |
| Neuroticism | 0.22 | -1.63 (3.50) | .643 | 0.00% |
| Agreeableness | 16.16 | -19.63 (4.88) | < .001 | 46.97% |
| Conscientiousness | 0.05 | -1.42 (6.36) | .823 | 0.00% |
| Openness | 0.14 | 1.28 (3.36) | .701 | 0.00% |

*Note*: The *Q_m_* tests whether the moderator explains any heterogeneity; the *B* is the coefficient resulting for meta-regression; the *p* is the significance of *Q_m_* and *B*; *R*^2^ is the amount of heterogeneity accounted for by the moderator.

Table 3. Multivariate analysis by including all five personalities in a single model.

| Moderator | *B* (*SE*) | *Z* | *p* |
| --- | --- | --- | --- |
| Intercept | 20.02 (7.18) | 2.79 | .005 |
| Extraversion | 15.64 (5.84) | 2.68 | .007 |
| Neuroticism | -3.91 (3.30) | -1.18 | .236 |
| Agreeableness | -36.17 (6.61) | -5.47 | <.001 |
| Conscientiousness | -0.53 (4.32) | -0.12 | .902 |
| Openness | -3.41 (3.34) | -1.02 | .308 |
